# The gut-kidney axis in chronic kidney disease: a vicious cycle of microbial dysbiosis and uremic toxin accumulation

**DOI:** 10.3389/fimmu.2026.1801912

**Published:** 2026-05-08

**Authors:** Ruohan Guan, Jiaxuan Peng, Qianxi Gu, Jiayi Xu, Jiaxin Zhang, Hamdalatu Adamu, Qiao Yang, Lu Wang, Gang Cao

**Affiliations:** 1 The First Affiliated Hospital of Zhejiang Chinese Medical University (Zhejiang Provincial Hospital of Chinese Medicine), Zhejiang Chinese Medical University, Hangzhou, China; 2 School of Pharmacy, Zhejiang Chinese Medical University, Hangzhou, China; 3 Jinhua Institute, Zhejiang Chinese Medical University, Hangzhou, China

**Keywords:** CKD, gut microbiota, gut-kidney axis, immunity, inflammation, uremic toxins

## Abstract

The progression of Chronic Kidney Disease (CKD) is governed by a pathogenic interplay between the host and the gut microbiota, a relationship encapsulated in the gut-kidney axis. This review synthesizes current knowledge on gut microbial alterations, metabolic reprogramming, and immunopathological mechanisms in CKD. Patients with CKD exhibit reduced microbial diversity, depletion of beneficial butyrate−producing bacteria, and enrichment of pathogenic taxa harboring urease, tryptophanase, and tyrosine phenol−lyase. These compositional shifts drive overproduction of protein−bound uremic toxins (indoxyl sulfate, p−cresyl sulfate, phenylacetylglutamine) and non−protein−bound toxins (trimethylamine N−oxide) via tryptophan, tyrosine, and arginine metabolic pathways. Mechanistically, uremic toxins disrupt intestinal tight junctions, promote endotoxin translocation, and trigger systemic inflammation. At the renal level, they induce direct cytotoxicity, endothelial dysfunction, oxidative stress (via NF−κB, MAPK, AhR signaling), and activation of both innate and adaptive immune responses. These processes converge to promote epithelial–mesenchymal transition, myofibroblast activation and renal fibrosis. Importantly, these elements form a self-sustaining vicious cycle: declining renal function leads to toxin buildup, which further accelerates CKD progression and its systemic complications. By comprehensively elucidating this bidirectional communication, we underscore the promising therapeutic strategy of suppressing uremic toxin generation or replenishing beneficial metabolites such as short−chain fatty acids to disrupt this cycle, thereby opening avenues for decelerating the advancement of CKD.

## 1 Introduction

Chronic kidney disease (CKD) is a global public health issue with a complex pathogenesis, prolonged course, and frequent complications. In recent years, with advances in microbiome research, the association between gut microbiota and CKD has become a hotspot. Investigating this relationship is crucial for understanding the role of gut microbiota and their metabolites in CKD progression and for developing effective therapeutic strategies. Numerous clinical studies indicate that CKD patients commonly exhibit significant alterations in the structure and function of their gut microbiota, characterized by reduced diversity, enrichment of pathogens, and a decrease in beneficial bacteria. For instance, Laiola M’s (1) research taxonomically frequently observed increased abundances of *Enterocloster*, *Hungatella*, *Desulfovibrio*, *Alistipes*, *Flavonifractor*, *Victivalles*, *Oscillibacter* and *Intestinimonas* in CKD patients, while lower abundances of *Faecalibacterium*, *Romboutsia*, *Anaerobutyricum* and *Intestinibacter* were noted. Furthermore, animal studies by Yao et al. (2) revealed that gut microbes such as *Bacteroides* and *Paraprevotella* increase the production of uremic toxins and promote CKD development. These findings illuminate the interaction between gut microbial dysbiosis and CKD.

In recent years, with deepening research, the gut microbiota-centric gut-kidney axis has emerged as a compelling framework for understanding the interconnected pathophysiology of CKD. This concept emphasizes the intricate bidirectional communication between the gastrointestinal tract and the kidneys (3). Uremic toxins (*e.g*., Indoxyl Sulfate, p-Cresyl Sulfate) play a key role in this process. Recent extensive research, such as that by Wakino S (4) and colleagues building on prior findings describing the gut-kidney axis, indicated that specific amino acids like tryptophan and tyrosine can generate uremic toxins (5). These produced uremic toxins exert multiple pathophysiological effects by activating different signaling pathways, including direct cytotoxicity, induction of endothelial dysfunction, triggering oxidative stress, promoting inflammatory responses, and immune activation, thereby accelerating the process of renal fibrosis and ultimately leading to CKD progression. Notably, the regulation of the gut-kidney axis is bidirectional: on one hand, gut microbiota dysbiosis promotes the accumulation of uremic toxins, accelerating renal function deterioration, while CKD-related internal environmental changes reciprocally affect gut microbiota structure, forming a vicious cycle; on the other hand, some beneficial metabolites produced by the gut microbiota can slow CKD progression. This complex interaction mechanism provides a theoretical basis for targeting the gut microbiota to intervene in CKD.

## 2 Gut microbial signatures and metabolic phenotypes in CKD patients

### 2.1 Altered microbial diversity and composition in CKD patients

The human gut microbial community consists of >100 trillion microbial cells, encompassing over 1000 different species of bacteria, viruses, and mycoplasmas (5), with bacteria being the most prevalent, collectively referred to as the gut microbiota. This gut microbiota includes symbiotic bacteria such as *Bifidobacterium* and *Lactobacillus*, conditional pathogens like *Enterobacter* and *Enterococcus*, and pathogenic bacteria such as *Staphylococcus (*6). These microbiotas primarily belong to 7 phyla, with Firmicutes, Bacteroidetes, and Actinobacteria being predominant (7). Under normal conditions, this gut microbiota maintains a relatively stable dynamic equilibrium, thereby sustaining various normal physiological activities in the human body. Related research is typically conducted at taxonomic levels such as phylum, family, genus, and species.

With the progressive decline of renal function, the diversity of the gut microbiota in CKD patients undergoes significant changes, characterized by reduced alpha diversity and increased unevenness in microbial beta diversity. These changes in microbial diversity have been consistently validated across both human and animal levels. At the human level, a systematic review including 25 studies with 1436 CKD patients showed that ESKD patients had reduced alpha diversity of gut microbiota, while beta diversity was significantly higher compared to healthy controls (8). This phenomenon is further validated in animal models. In a mouse model experiment, Liu et al. (9), using a sham operation group, a unilateral nephrectomy mouse model was gavaged with CMC-Na (UNx group), and a unilateral nephrectomy mouse model gavaged with adenine and potassium oxonate suspension (UNx+HPD group) to simulate CKD, found that the UNx and UNx+HPD groups had reduced alpha diversity, and the beta diversity of both groups was clearly separated from the sham operation group. While the above-described taxonomic shifts in gut microbiota of CKD patients were robustly documented across multiple studies, several critical caveats warrant consideration. For example, the heterogeneity of reported changes—particularly the contradictory findings regarding *Prevotella* (decreased at species level for *P. copri* yet increased at genus level)—highlights a fundamental limitation of 16S rRNA sequencing: insufficient phylogenetic resolution. Many genera contain species with opposing functional roles, and bulk taxonomic averaging may obscure clinically relevant microbial signatures. This suggests that functional metagenomics, rather than taxonomic profiling alone, is urgently needed to identify conserved microbial metabolic pathways that directly produce uremic toxins.

Beyond diversity, the compositional structure of the gut microbiota is also profoundly altered in CKD. Significant differences in the abundance of 190 bacterial OTUs were observed between ESRD and healthy individuals (10). Studies based on metagenomic and 16S rRNA sequencing have further revealed significant dysbiosis in the gut microbiota of patients with CKD across multiple taxonomic hierarchies, including phylum, family, and genus. At the phylum level, there was an increase in the abundance of Proteobacteria and Bacteroidetes, while a decrease was observed in the populations of Actinobacteria and Firmicutes (11). At the family level, current clinical evidence indicates that patients with CKD exhibit enrichment of specific bacterial taxa harboring distinct metabolic enzyme systems in the gut compared to healthy individuals. For instance, certain bacterial families co-express urease, indole and p-cresol forming enzymes, including Clostridiaceae and Enterobacteriaceae; others co-harbor urease and uricase, such as Cellulomonadaceae, Dermabacteraceae, Xanthomonadaceae, Polyangiaceae, Pseudomonadaceae, and Micrococcaceae; some possess urease exclusively, including Alteromonadaceae, Halomonadaceae, Methylococcaceae, and Moraxellaceae; while Verrucomicrobiaceae solely encoded enzymes for indole and p-cresol formation. Furthermore, the abundances of Lachnospiraceae, Ruminococcaceae, Eggerthellaceae, and Thiothrix were elevated in CKD, whereas those of Lactobacillaceae, Veillonellaceae, Bacteroidaceae, Prevotellaceae, and Bifidobacteriaceae reduced (6, 7, 12). At the species/genus level, species enriched in ESRD patients included *Eggerthella lenta*, *Flavonifractor* spp. (mainly *F. plautii*), *Alistipes* spp. (mainly *A. finegoldii* and *A. shahii*), *Ruminococcus*, and *Fusobacterium*. Decreased species included *Prevotella* (mainly *P. copri*), *Clostridium*, and butyrate-producing bacteria represented by *Roseburia* spp., *Faecalibacterium prausnitzii*, and *Eubacterium rectale (*13). Notably, *P. copri* was reduced at the species level, the genus *Prevotella* as a whole was reported to be enriched in another CKD cohort (11), suggesting that other species within this genus may increase in abundance. The same report showed that enriched genera in CKD include *Klebsiella*, *Enterobacter*, *Streptococcus*, *Turicibacter*, *Prevotella*, *Ruminococcus*, and *Mogibacterium*, while depleted genera include *Akkermansia*, *Faecalibacterium*, *Coprococcus*, *Butyricicoccus*, *Butyricimonas*, *Oscillospira*, *Adlercreutzia*, *Dorea*, *Bifidobacterium*, and *Collinsella (*11). The reported changes involve dozens of genera and hundreds of OTUs, yet no single “CKD-specific microbial signature” has emerged that reliably discriminates disease stage or predicts progression across independent cohorts. This lack of reproducibility may stem from confounding factors rarely controlled for: dietary intake (especially protein and fiber), medication use (antibiotics, phosphate binders, proton pump inhibitors), dialysis modality, and residual renal function. Without rigorous adjustment for these variables, many reported associations may be confounded rather than causal.

Overall, the dynamic balance of the intestinal flora in CKD is disrupted, specifically manifested by a decrease in beneficial bacteria and an abnormal increase in pathogenic and conditional pathogenic bacteria (6), as well as an increase in the number of aerobic and anaerobic microorganisms (14). The diversity and composition of the gut microbiota (**Table 1**) undergo significant changes, leading to an imbalance in the intestinal microenvironment. The current descriptive catalog of CKD-associated microbiota alterations provides an essential foundation, but the field must now prioritize functional validation, causality testing through microbiota transplantation or defined consortia, and integration of multi-omics to identify actionable therapeutic targets. Without such mechanistic depth, taxonomic patterns alone will not translate into precision microbiome therapies for CKD patients.

**Table 1:** Level changes in gut microbiota in CKD.

Phylum	Family	Genus	Species	Cite
Proteobacteria	Alteromonadaceae↑			(7)
	Halomonadaceae↑			(7)
	Methylococcaceae↑			(7)
	Moraxellaceae↑			(7)
	Xanthomonadaceae↑			(7)
	Pseudomonadaceae↑			(7)
	Enterobacteriaceae↑	*klebsiella*↑		(6, 7, 11, 12)
		*Citrobacter*↑		(6, 7, 12, 15)
		*Enterococci*↑		(6, 7, 12)
		*Enterobacter*↑		(6, 7, 11, 12)
Firmicutes	Clostridiaceae(ESRD)↑(CKD)↓	*Clostridium*↓		(6, 7, 10)
	Ruminococcaceae↑	*Ruminococcus*↑		(6, 10)
	Lachnospiraceae↑	*Roseburia*↓		(6, 10, 12)
		*Coprococcus*		(6, 11, 12)
		*Dorea*↓		(6, 11, 12)
		*Hungatella*↑		(6, 12)
		*Lachnospira*↓		(6, 12)
		*Agathobacter*↓		(6, 12, 16)
		*Anaerostipes*↓		(6, 12, 17)
		*Eubacterium*↓	*Eubacterium rectale*↓	(6, 10, 12, 16, 17)
		*Blautia*↑		(6, 12)
		*Mogibacterium*↑		(6, 11, 12)
	Streptococcaceae↑	*Streptococcus*↑		(11, 15)
	Lactobacillaceae↓	*Lactobacillus*↓		(6)
	Oscillospiraceae	*Flavonifractor*↑		(10)
		*Butyricicoccus*↓		(11)
		*Oscillospira*↓		(6)
		*Faecalibacterium*↓	*Faecalibacterium prausnitzi*↓	(6, 10–12, 16, 17)
	Erysipelotrichaceae	*Coprobacillus*↑		(17)
Actinobacteria	Cellulomonadaceae↑			(7)
	Dermabacteraceae↑			(7)
	Micrococcaceae↑			(7)
	Bifidobacteriaceae	*Bifidobacterium*↓		(6, 11)
	Actinomycetaceae	*Actinomyces*↓		(16)
	Eggerthellaceae↑	*Eggerthella*	*Eggerthella lenta*↑	(10, 12)
	Coriobacteriaceae	*Adlercreutzia*↓		(11)
		*Collinsella*↓	*Collinsella intestinalis*↑	(11, 12)
Myxococcota	Polyangiaceae↑			(7)
Verrucomicrobia	Verrucomicrobiaceae↑			(7)
	Akkermansiaceae	*Akkermansia*↓		(11)
Bacteroidetes	Rikenellaceae	*Alistipes*↑		(10)
	Butyricimonaceae	*Butyricimonas*↓		(11)
	Bacteroidaceae↓	*Bacteroides*↓		(6)
	Muribaculaceae↓			(12)
	Prevotellaceae↓	*Prevotella*↓		(6, 10)
	Turicibacteraceae	*Turicibacter*↑		(11)
Fusobacteriota	Fusobacteriaceae	*Fusobacterium*↑		(10, 12)
Acidobacteriota	Solibacteraceae	*Candidatus-Solibacter*↑		(15, 16)

### 2.2 Metabolic dysfunction and uremic toxin genesis in CKD

Uremic toxins can be classified into three categories based on their physicochemical properties: Class 1 includes small, non-protein-bound, water-soluble molecules (molecular weight < 500 Da), Class 2 includes middle molecules (molecular weight > 500 Da), and Class 3 includes protein-bound molecules (18). The generation of these toxins relies on precursor substances produced by gut microbial metabolism. Therefore, studying the bacteria that produce these precursors can enhance the understanding of the relationship between uremic toxins and CKD.

Precursors for water-soluble uremic toxins include ammonia, a precursor of urea, and trimethylamine (TMA), a precursor of trimethylamine N-oxide (TMAO). Bacteria containing urease, such as Pseudomonadaceae, are producers of urea precursors (19). Urea can enter the gastrointestinal tract and be decomposed by gut bacteria containing urease into ammonia and CO_2_; these bacteria then utilize ammonia to produce amino acids and peptides (20). *Escherichia*, *Klebsiella*, *Kluyvera*, and *Citrobacter*, due to carrying specific genes for enzymes responsible for TMA synthesis like CutC, CntA, GrdH, and TorA, participate in TMA synthesis and are thus precursor-producing bacteria for TMAO (21).

Precursors for protein-bound uremic toxins include indole (precursor for IS), p-cresol (precursor for pCS), and phenylacetic acid (precursor for phenylacetylglutamine). IS is a tryptophan-derived uremic toxin. Tryptophan is metabolized by gut bacteria containing tryptophanase, such as *Escherichia coli*, into indole (6). Liang et al. (22), using a weaned piglet model, found that at the genus level, in the 0.2% tryptophan group, *Anaerotruncus* and *Treponema* were the most abundant bacteria, while in the 0.4% tryptophan group, *Succinivibrio* and *Tissierella* were relatively abundant; these can be considered indole-producing bacteria. The pCS is a product of phenylalanine and tyrosine metabolism. Studies indicate that among gut bacteria producing phenolic compounds, *Eubacterium*, *Clostridium*, *Fusobacterium*, *Anaerococcus*, *Ruminococcus*, and *Roseburia* are closely associated with the generation of p-cresol (23). Phenylacetylglutamine is a colonic microbial metabolite derived from phenylalanine via phenylacetic acid. Its precursor-producing gut microbes primarily belong to the phyla Bacteroidetes, Firmicutes, and Proteobacteria (24).

**Figure 1:**
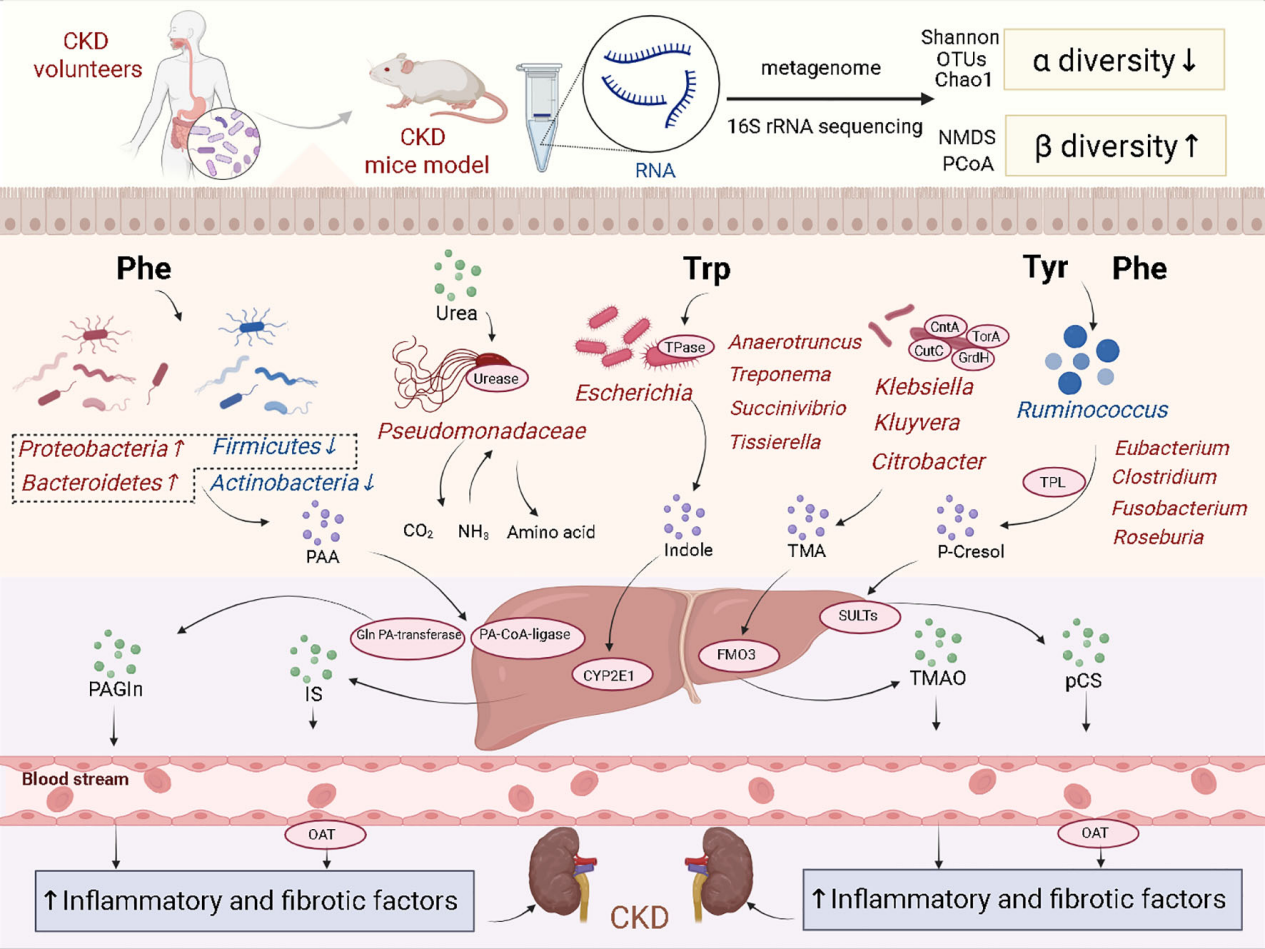
The gut microbiota and uremic toxins in CKD. The diversity of the gut microbiota in CKD decreases. Amino acids are converted into uremic toxins precursor under the action of *Klebsiella, Ruminococcus* and *Escherichia*. These precursors are converted into uremic toxins by the liver and accumulate in the body, thereby triggering kidney inflammation and fibrosis.

Most of these uremic toxin precursor-producing bacteria show high expression in CKD, also suggesting that the impact of gut microbiota on CKD progression is closely related to uremic toxins (**Figure 1**).

## 3 Pathophysiological mechanisms of the gut-kidney axis

### 3.1 The proposal of gut-kidney axis

**Figure 2:**
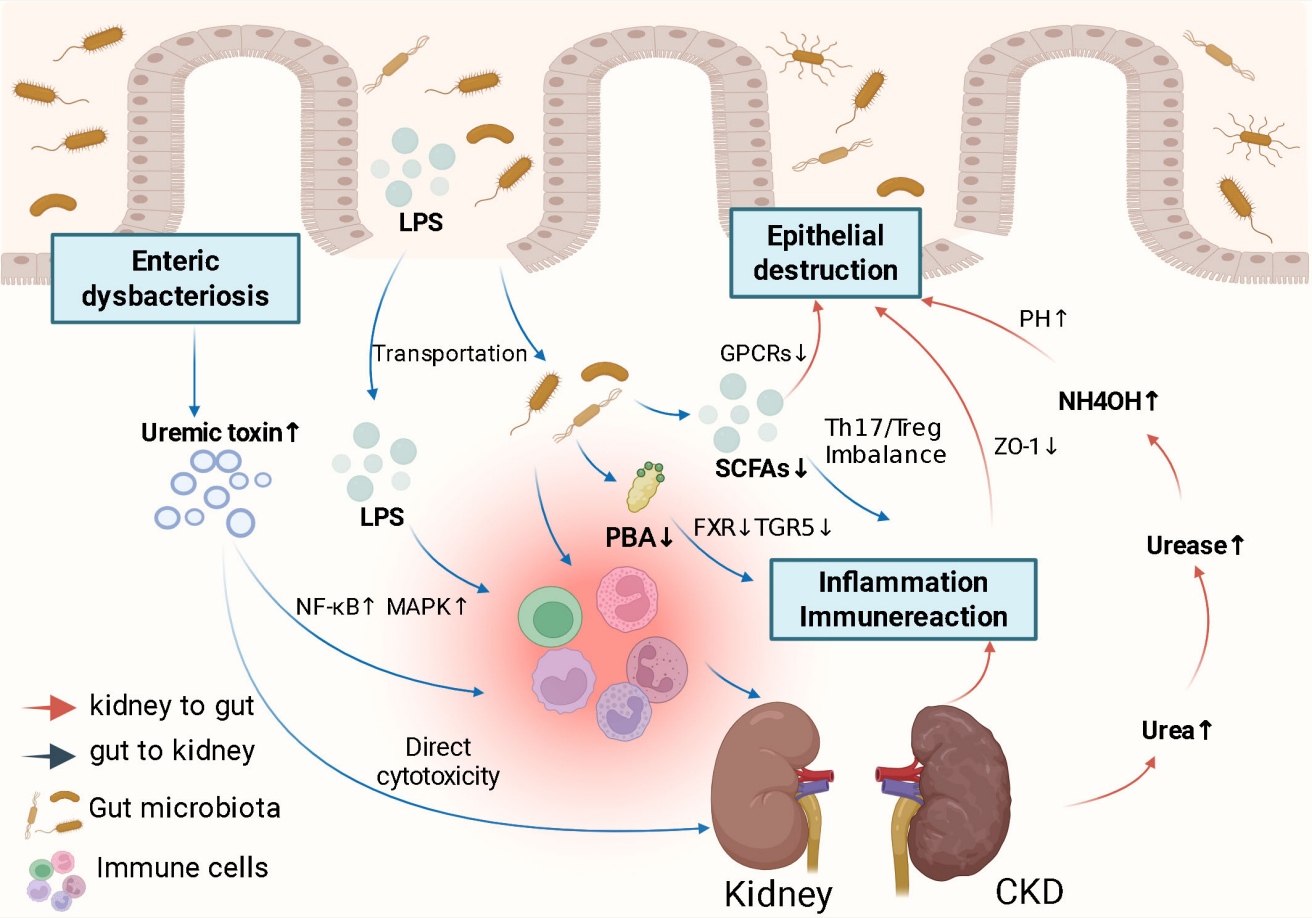
Gut-kidney axis. The pathways of the gut-kidney axis are illustrated above, functioning through gut microbial dysbiosis, disruption of epithelial tight junctions and gut barrier damage, LPS and bacterial translocation, accumulation of gut metabolites, inflammatory response, and immune response—namely, the “intestinal mucosal damage-dysbiosis-inflammatory response” cycle—ultimately leading to continuous deterioration of gut barrier function and thereby accelerating CKD progression.

In recent years, increasing evidence indicates a close connection between the kidneys and the gut microbiota, known as the gut-kidney axis, a concept proposed by scholars as early as 2011 (6). The gut-kidney axis is a bidirectional pathway between gut bacteria and the kidneys, formed through metabolites, inflammatory mediators, and immune signals (24, 25). Under CKD conditions, impaired intestinal barrier function, abnormal microbial metabolites, and gut dysbiosis can persistently activate the intestinal immune system. This will lead to the release of pro-inflammatory cytokines into systemic circulation, and subsequently act on the kidneys and exacerbate renal fibrosis and functional decline. Concurrently, the uremic internal environment resulting from impaired renal function further damages the intestinal immune barrier and aggravates gut microbial disturbances, thereby establishing a self-perpetuating vicious cycle (**Figure 2**).

This conceptual framework transcends the conventional view of renal pathology in isolation, redefining the kidney and the gut as an integrated dialogic system. It highlights the gut as the initiation site of chronic inflammation, while the kidney serves not only as a target organ but also as an amplifier of inflammatory responses. Consequently, the gut–kidney axis can be understood as a unified framework, mediated by immune and metabolic pathways, that links gut immunity with microbial metabolism, thereby opening new avenues for immune-modulatory strategies beyond traditional pharmacological approaches (26). However, the substantial inter-individual variability in gut microbiota composition and the elusiveness of precise mechanisms considerably hinder the translation of this knowledge into clinical therapeutics.

### 3.2 Gut barrier dysfunction and endotoxin translocation

The gut barrier is a complex protective system comprising physical, chemical, microbial, and immune barriers, with tight junction proteins being a critical element (27).

Under normal conditions, protected by tight junction proteins, the gut barrier prevents the passage of intestinal pathogens (28). However, as renal excretory function declines in CKD, urea cannot be promptly excreted, leading to its accumulation in the body. This urea is metabolized by gut bacteria, producing NH_3_ which is hydrolyzed to NH_4_OH, raising the intestinal pH. This alkaline environment disrupts the connections of tight junction proteins, increases intestinal permeability, and induces apoptosis of intestinal epithelial cells, damaging the intestinal mucosa (6, 29). Damage to the intestinal mucosa can lead to bacterial translocation, causing dysbiosis of the gut microbiota in CKD and inducing local inflammation, which in turn further aggravates mucosal damage (29). Impairment of the gut barrier allows endotoxins, normally confined to the intestinal lumen, to translocate into the portal vein. Endotoxins exacerbate intestinal inflammation and impair the function of the intestinal mucosal barrier.

This multifactorial, interactive pathological process forms a cycle of “intestinal mucosal damage-dysbiosis-inflammatory response”, ultimately leading to the continuous deterioration of gut barrier function in CKD and accelerating CKD progression.

### 3.3 Metabolic pathways of uremic toxins

#### 3.3.1 Sources of uremic toxins

**Figure 3:**
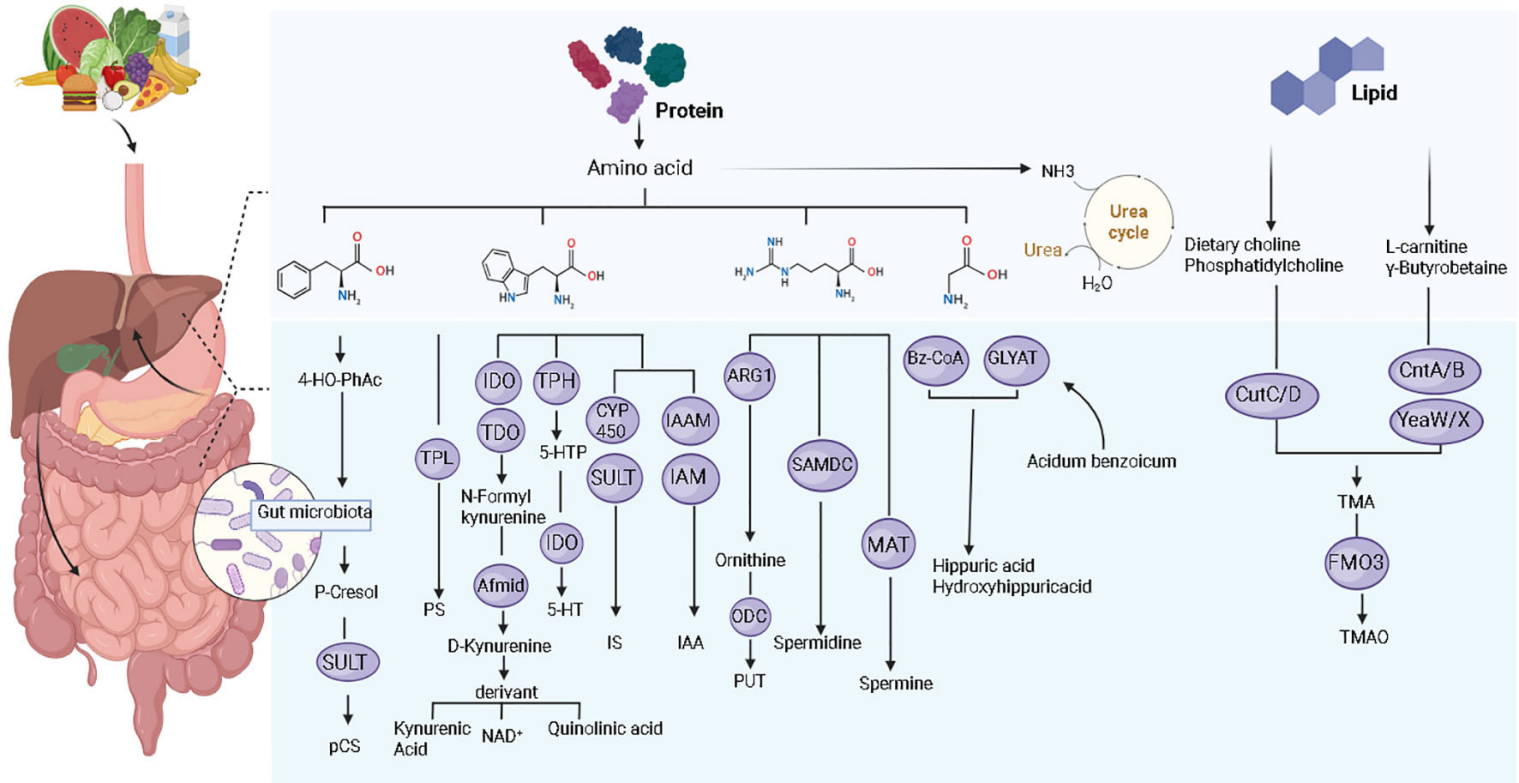
Metabolic pathways of gut-derived uremic toxins. Primarily generated by the gut microbiota metabolizing dietary amino acids (such as tryptophan and tyrosine). These microbial metabolites are then transported via the portal vein to the liver, where they undergo further transformation by various enzyme systems within hepatocytes, ultimately forming uremic toxins such as IS and pCS.

Amino acids like choline, tyrosine, phenylalanine, and tryptophan can serve as precursor substances during bacterial metabolism in the gut (5). These are converted into metabolic intermediates by the microbiota, which are then further transformed into uremic toxins by enzyme systems in the host liver or intestinal epithelial cells (29).These uremic toxins are closely related to the generation if inflammation (**Figure 3**).

Protein-bound uremic toxins mostly originate from the gut and are products of the breakdown of aromatic amino acids by gut microbiota (30). These protein-bound uremic toxins include indole toxins, phenolic toxins, polyamine toxins, Hippurates, and AGEs (31). Common indole toxins include IS and IAA. The production of IS is related to dietary tryptophan. Tryptophan is converted to indole by gut microbiota, and then further metabolized to IS in the liver via cytochrome P450 enzymes and sulfotransferases (29). Similarly, IAA synthesis is achieved through the action of tryptophan monooxygenase and indole-3-acetamide hydrolase on tryptophan (32). Common phenolic toxins include pCS and PS, both related to tyrosine. The pCS is metabolically produced in the gut and liver. Tyrosine is converted to 4-hydroxyphenylacetic acid, which then forms p-cresol via decarboxylation by bacteria like *Clostridium difficile*. Most p-cresol is absorbed and catalyzed by sulfotransferases to generate pCS (29). PS production first involves the decomposition of tyrosine into phenol by gut bacteria expressing tyrosine phenol-lyase, which is then metabolized in the liver to ultimately synthesize PS (33). Polyamine uremic toxins are three small aliphatic amines (putrescine, spermidine, and spermine) derived from dietary intake, gut microbial metabolism, and host biosynthesis (31). Arginine is catalyzed by arginase 1 (ARG1) to produce ornithine, which is decarboxylated by ornithine decarboxylase to generate putrescine (34). Spermine and spermidine are formed by the transfer of aminopropyl groups onto putrescine or spermidine, catalyzed by S-adenosylmethionine decarboxylase and specific transferases, respectively (35). Whether spermidine and spermine belong to uremic toxins remains controversial, as some studies indicate that their serum levels are lower in CKD patients compared to healthy controls (31). Hippurates, such as hippuric acid and hydroxyhippuric acid, are synthesized in the mitochondrial matrix of hepatocytes through a two-step enzymatic process. First, benzoic acid is converted to benzoyl-CoA by benzoyl-CoA synthetase. Subsequently, benzoyl-CoA:glycine N-acyltransferase catalyzes the condensation of benzoyl-CoA with glycine to form hippuric acid (31). AGEs are a heterogeneous group of compounds formed through the non-enzymatic glycosylation of proteins, lipids, or nucleic acids, and can originate from exogenous sources (36).

Non-protein-bound uremic toxins are divided into free water-soluble low molecular weight uremic toxins and middle molecule uremic toxins. Free water-soluble low molecular weight uremic toxins include TMAO, ADMA, and urea. Dietary choline and phosphatidylcholine are converted to TMA by the TMA lyase complex CutC/D; L-carnitine and γ-butyrobetaine are converted to TMA by the TMA lyase complexes CntA/B and YeaW/X. In the liver, TMA is converted to TMAO catalyzed by FMO3 (31). ADMA, a non-proteinogenic amino acid, is produced by the post-translational methylation of arginine side chains in proteins by the protein arginine methyltransferase family (37). Urea production occurs through the ornithine cycle, where ammonia derived from protein catabolism is converted into urea (20). Middle molecule uremic toxins include TNF-α, IL-6, etc. These are pro-inflammatory cytokines produced by immune cells (*e.g*., T lymphocytes, B lymphocytes, macrophages) and other cells (*e.g*., vascular endothelial cells, renal tubular epithelial cells) (30). This comprehensive listing of toxin sources, while informative, conflates host−derived cytokines with microbial metabolites, includes molecules paradoxically decreased in CKD (*e.g*., spermidine), and lacks quantitative prioritization. Distinguishing accumulating solutes from causative toxins is essential for clinical translation.

#### 3.3.2 Tryptophan metabolic pathway

Tryptophan is an essential aromatic amino acid. Although tryptophan is the least abundant in proteins and cells, it is a precursor for numerous host metabolites (38).

Tryptophan metabolism is closely linked to the production of indole uremic toxins. Common indole uremic toxins, such as IS, IAA, I3A, ILA, IPA, and tryptamine, all originate from tryptophan metabolism.

Tryptophan metabolism occurs via three main pathways. The first is the kynurenine pathway, the major route of tryptophan metabolism. In this pathway, tryptophan is catalyzed by IDO1, IDO2, and tryptophan 2, 3-dioxygenase to generate N-formylkynurenine, which is then converted to kynurenine by arylformamidase. Subsequently, kynurenine can be further metabolized to produce various derivatives, including kynurenic acid, xanthurenic acid, picolinic acid, quinolinic acid, and NAD+ (39). The second metabolic pathway is the serotonin pathway, where tryptophan is first catalyzed by tryptophan hydroxylase to form 5-hydroxytryptophan, which is then further metabolized by aromatic L-amino acid decarboxylase to 5-hydroxytryptamine (38). The third metabolic pathway is the indole pathway, where tryptophan is converted to indole by gut bacteria. The generated indole is further metabolized into indole derivatives, i.e., indole uremic toxins (40). Increasing research shows that different gut bacteria participate in the synthesis of these toxins. Tryptophan can be metabolized to I3A by *Lactobacillus*, to ILA by *Bifidobacterium* and *Bacteroides*, to IAA by *Bifidobacterium*, *Bacteroides*, and *Clostridium*, to IPA by *Peptostreptococcus*, and tryptamine can be produced from tryptophan by *Clostridium sporogenes* and *Ruminococcus (*39). These toxins accelerate CKD progression and promote renal fibrosis.

#### 3.3.3 Tyrosine metabolic pathway

Phenylalanine is an essential amino acid that humans cannot synthesize, while tyrosine, a conditionally essential amino acid, is produced only via the hydroxylation of phenylalanine. Both also originate from exogenous intake or endogenous protein breakdown (41).

In pathways starting with tyrosine, tyrosine can be reversibly converted to phenol by tyrosine phenol-lyase. Furthermore, tyrosine is reversibly catalyzed by phenylalanine dehydrogenase, tyrosine transaminase, and aromatic amino acid transaminase to produce hydroxyphenylpyruvate, which is then oxidized into homogentisate. Homogentisate can in turn, interact with p-hydroxyphenylacetate decarboxylase to form p-cresol. The generated p-cresol is then acted upon by aryl sulfotransferase and UDP-glucuronosyltransferase to produce p-cresyl sulfate and p-cresyl glucuronide, respectively (42). In pathways starting with phenylalanine, phenylalanine is reversibly converted to phenylpyruvate by phenylalanine, dehydrogenasebranched-chain amino acid transaminase, and aromatic amino acid transaminase. Phenylpyruvate can then be decarboxylated to phenylacetaldehyde or reduced to phenyllactate. Phenylacetaldehyde is further reversibly converted to phenylacetate by phenylacetaldehyde dehydrogenase (42). Plasma metabolomics studies have shown that *Akkermansia*, UCG-005, *Monoglobus*, and *Lachnospiraceae* NK4A136 group are negatively correlated with tyrosine, while *Lactococcus* and *Acidophilus* are positively correlated (9).

#### 3.3.4 Other metabolic pathways

Beyond the tryptophan and tyrosine metabolic pathways mentioned above, uremic toxins are also associated with other metabolic pathways.

Ornithine and arginine both contribute significantly to polyamine metabolism. Ornithine is related to the ornithine-polyamine synthesis pathway. Arginine primarily follows the deiminase route, a key diamine biosynthesis pathway prevalent in the human gut microbiome. Additionally, the arginine deiminase pathway is common in gut commensal bacteria such as *Enterococcus*, *Streptococcus*, *Clostridium*, *Lactococcus*, and *Lactobacillus (*43). In this metabolic network, ornithine decarboxylase, controlled by antizyme and antizyme inhibitor, plays a significant role (44). Polyamine accumulation is associated with cell proliferation, oxidative stress, and inflammatory responses, potentially promoting CKD progression.

The primary metabolic reaction related to AGEs is the non-enzymatic Maillard reaction, which begins with nucleophilic addition between reducing sugars and protein amino groups to form Schiff bases; these rearrange into Amadori products, then oxidize (often metal-catalyzed) to yield stable AGEs. Other metabolic pathways contributing to AGE formation include glucose autoxidation, lipid peroxidation, and the polyol pathway. In the polyol pathway, glucose is converted to sorbitol by aldose reductase and then to fructose by sorbitol dehydrogenase. Fructose metabolites can be further transformed into reactive dicarbonyl species, like glyoxal, methylglyoxal, and 3-deoxyglucosone, which act as key precursors that modify proteins and DNA, ultimately forming AGEs (45). The generated AGEs significantly impact the gut microbiota, characterized by an increase in *Bacteroidetes*, a decrease in *Firmicutes* abundance, and a reduced relative abundance of *Lactobacillus*, contributing to systemic inflammation in CKD (46).

#### 3.3.5 The interaction of metabolic pathways and immune circuits

The metabolic processing of uremic toxins does not occur as isolated biochemical reactions; rather, it is deeply integrated into the host’s immune-metabolic network through shared intermediates, cofactors, and enzymatic regulation (**Table 2**).

**Table 2:** Uremic toxin pathways and immune-pathological outcomes.

Metabolic Pathways	Uremia Toxin	Gut Microbiota	Key Enzyme	Immune Response Effect	Clinical pathological consequences
Tryptophan Metabolic Pathway	IS	*Escherichia coli*	CYP450 SULT	1.Activation of inflammasome:ROS/TXNIP/NLRP3 2.Signal pathways:Activation of AhR, NF-κB, MAPK (p38/JNK), TGF-β/Smad 3.Immune cells:Macrophage chemotaxis and activation, Upregulation of Th17/Treg imbalance	1.Kidney: EMT, Renal fibrosis, Podocyte injury 2.Vessels: Inducing endothelial dysfunction (VCAM-1/ICAM-1), Promoting vascular calcification 3.Intestine: Inhibiting HIF/IL-10/VEGF, Damaging intestinal barrier
	IAA	*Bifidobacterium* *Bacteroides* *Clostridium*	IAAM IAM	1.Signal pathway:Activation of the AhR/p38 MAPK/NF-κB pathway 2.Oxidative stress:Induction of COX-2 expression 3.Genotoxicity: Causing DNA damage in neutrophils	1.Kidney: Stimulate glomerular sclerosis 2.Mitochondria: Disrupting energy metabolism and the electron transport chain
	ILA	*Bifidobacterium Bacteroides*			
	I3A	*Lactobacillus*			
	IPA	*Peptostreptococcus*			
	Tryptamine	*Clostridium sporogenes* *Ruminococcus*			
	Kynurenine		IDO	1.Immune regulation:Activation of AhR, Regulation of Treg/Th17 balance	
	Quinolinic Acid		IDO	1.Immune regulation:Activation of AhR, Regulation of Treg/Th17 balance 2.Cytotoxicity: Induces lipid peroxidation and disrupts the phospholipid bilayer, Interfering with mitochondrial energy metabolism and the electron transport chain	1.Neuro/Immune: Imbalance of immune tolerance
Tyrosine Metabolic Pathway	pCS	*Lactococcus* *Acidophilus*	SULT	1.Signal pathways:Activation of TGF-β/Smad, RAAS, AhR 2.Immunosuppression: Inhibition of Th1 cells (IFN-γ), Suppression of macrophage antibacterial activity 3.Epigenetics: Upregulation of DNMT (DNA methyltransferase) expression	1.Kidney: Induces EMT and EndMT, Promotes fibrosis 2. Immunity: Inhibits proliferation of B cell precursors, Impairs cellular immune defense
	PS	*Lactococcus* *Acidophilus*	TPL	1.Signal pathways:Activation of TGF-β/Smad, RAAS, AhR 2.Immunosuppression: Inhibition of Th1 cells (IFN-γ), Suppression of macrophage antibacterial activity 3.Epigenetics: Upregulation of DNMT (DNA methyltransferase) expression	1.Kidney: Induces EMT and EndMT, Promotes fibrosis 2.Immunity: Inhibits proliferation of B cell precursors, Impairs cellular immune defense
	PAGIn	Bacteroidetes Firmicutes Proteobacteria		1.Direct toxicity:Podocyte hypertrophy and fusion of foot processes	1.Kidney: Aggravate proteinuria, Damage the filtration barrier
Choline/Carnitine Metabolic Pathway	TMAO		FMO3	1.Inflammasome: Activation of ROS-TXNIP-NLRP3 2.Immune Adhesion: Upregulation of VCAM-1, promoting monocyte adhesion 3.Signaling Pathway: Upregulation of TLR4 gene expression 4.Cytotoxicity: Inhibits mitochondrial respiration (OXPHOS)	1.Cardiovascular: Promotes atherosclerosis
Arginine/Ornithine Metabolic Pathway	Putrescine Spermidine Spermine	*Enterococcus* *Streptococcus* *Clostridium* *Lactococcus* *Lactobacillus*	ARG1	1.Immune-metabolic switch:Elevated ARG1 activity inhibits NO production, Drives M2 macrophage polarization. 2.Cell proliferation:Promotes cell proliferation, Oxidative stress.	1.Bidirectional effect:Tissue repair, Excessive accumulation is associated with proliferative lesions in CKD
Maillard reaction Polyol pathway	AGEs			1.Signal pathway: Activation of the RAGE/NF-κB axis	1.Systemic: Aggravate systemic microinflammation 2.Vascular: Promote endothelial stiffness and hardening

The metabolic pathway of tryptophan through the kynurenine pathway is highly coupled with immune system activity. The initial conversion of tryptophan is catalyzed by IDO, the key rate-limiting enzyme in this pathway. The expression and enzymatic activity of IDO can be significantly upregulated by pro-inflammatory cytokines such as TNF-α, IL-6, and IFN-γ (47), and contribute to the regulation of the Treg/Th17 balance (48), thereby linking immune activation status closely with kynurenine production, thereby linking immune activation closely with kynurenine production. Kynurenine acts not only as a negative allosteric modulator of the α7 nicotinic acetylcholine receptor but also activates G protein-coupled receptors and the AhR. Among these, AhR activation plays a particularly critical role in immune modulation (47). In tyrosine metabolism, the final metabolite pCS, generated through the intestinal microbiota conversion and hepatic metabolism, inhibits immune responses of Th-1 cells and macrophages. Meanwhile, studies have demonstrated that pCS can activate the AhR signaling pathway (49), which interacts with AhR-mediated immune-regulation in the kynurenine pathway.

The arginine-ornithine pathway constitutes a core metabolic switch for immune regulation. ARG and NOS2 compete for the substrate L-arginine, determining the direction of the immune response: NOS2 generates NO to mediate pro-inflammatory reactions, while ARG produces ornithine, which is then used to synthesize polyamines and proline, supporting anti-inflammatory, repair and proliferation. Different immune cells (such as M1/M2 macrophages, T cells, NK cells, and dendritic cells) rely on this metabolic balance to achieve functional specialization. Notably, dendritic cells regulate arginine metabolism through IDO1, thereby enhancing the immune suppression program, which forms a significant metabolic crosstalk with the aforementioned tryptophan metabolic pathway (50).

In summary, the metabolic pathways of uremic toxins and the immune circuits are intertwined at multiple nodes. These close connections suggest that merely eliminating toxins may not be sufficient to interrupt the malignant progression of CKD. It is necessary to actively intervene in the key nodes of the immune-metabolic network while eliminating toxins to effectively regulate chronic inflammation and immune disorders.

### 3.4 Bidirectional balance of gut metabolites in CKD progression

Gut microbial metabolites exert bidirectional regulatory effects on renal function, and their balance determines the progression trajectory of CKD. On the one hand, some beneficial gut metabolites, such as SCFAs, play roles in maintaining the gut barrier, antioxidant defense, and inhibiting fibrosis; primary bile acids have immune-modulatory effects. On the other hand, uremic toxins produced by gut bacteria during protein fermentation promote systemic inflammation and kidney injury (51).

As CKD progresses, the impaired filtering kidneys reduce the clearance of uremic toxins, creating a toxic cycle of the aforementioned metabolic imbalances, exacerbating renal and systemic damage (51). These uremic toxins increase in the bloodstream, inducing inflammation in kidney tissue, damaging the kidneys, and accelerating CKD progression (52). Experiments have shown that in animal models, uremic toxins can generate various inflammatory factors, thereby causing oxidative stress responses leading to renal tubular damage (29). Besides affecting the kidneys, uremic toxins also contribute to cardiovascular complications in CKD, impacting their prognosis (29).

The role of SCFAs slowing the progression of CKD is increasingly being elucidated, encompassing the suppression of inflammatory responses, inhibition of oxidative stress, modulation of autophagy, enhancement of energy metabolism, and regulation of immune pathways (53). At the immunological level, they modulate inflammatory responses, suppress oxidative stress, preserve intestinal epithelial barrier integrity, and participate in energy metabolism by activating GPCRs such as GPR41 and GPR43 (54). SCFAs facilitate the expansion and functional enhancement of Treg, thereby suppressing the release of pro-inflammatory cytokines such as TNF-α and IL-6. Furthermore, microbiota-derived SCFAs promote immune homeostasis by modulating the balance between proinflammatory Th17 cells and Treg (55). However, in CKD, diminished levels of SCFA-producing bacteria disrupt SCFA metabolism, resulting in decreased beneficial metabolites in the gut and consequently impairing their protective functions(**Figure 2**). Bile acid metabolism is a key link in regulating intestinal homeostasis. Primary bile acid, as an important signaling molecule, can restore intestinal barrier integrity and exert immunomodulatory effects by activating FXR and TGR5 (56). When bile acid metabolism is perturbed, with a decrease in primary bile acids, dysregulation of bile acid metabolism further affects systemic metabolic homeostasis and renal function. The excessive accumulation of harmful metabolites and the insufficiency of beneficial metabolites lead CKD into a vicious cycle of accelerated progression. Therefore, restoring the bidirectional balance of intestinal metabolites is expected to become a new strategy for the treatment of CKD.

## 4 Mechanisms of renal injury induced by uremic toxins

### 4.1 Direct cytotoxicity-induced immune initiation

In the progression of CKD, uremic toxins directly induce cellular damage by disrupting structural integrity, causing abnormal epigenetic modifications, and interfering with energy metabolism. This process is believed to further activate immune responses, thereby accelerating disease progression.

From the kidney to the gut, the direct cytotoxicity of uremic toxins can disrupt the integrity of intestinal epithelial cells. Uremic toxins like quinolinic acid can induce lipid peroxidation, destabilizing the phospholipid bilayer of cell membranes (31), leading to increased membrane permeability, cellular dysfunction, and even apoptosis or necrosis, thereby disrupting cellular integrity. Similarly, p-cresol (PC) can affect the cell cycle kinetics of HT29 and Caco-2 cells (57). Furthermore, studies by Marta Idziak et al. showed that uremic concentrations of PC and IS inhibit the metabolic activity and proliferation of mesenchymal stem cells (MSCs) and, although not causing apoptosis, disrupt the cell membrane (58). This discovery is not a classical form of cell death, and it remains questionable whether it can effectively trigger downstream immune cascade reactions.

From the gut to the kidney, the direct cytotoxicity of uremic toxins affects renal podocytes and interferes with energy metabolism. Phenylacetylglutamine and phenyl sulfate exhibit podocyte toxicity, causing podocyte hypertrophy and foot process effacement (7, 29). Uremic toxins disrupt epigenetic modifications through mechanisms such as DNA methylation; for instance, p-cresol has genotoxic effects, causing DNA damage (57). The pCS also increases the expression of DNA methyltransferase 1, 3a, and 3b isoforms. Additionally, certain uremic toxins like IAA can damage the DNA of human neutrophils (57), and both IAA and pCS upregulate mRNA expression (23). The aforementioned DNA damage and alterations in gene epigenetic regulation, including aberrant protein modifications, can orchestrate gene expression profiles to modulate immune response (59). Uremic toxins can interfere with energy metabolism by affecting mitochondria. Indole uremic toxins such as IAA, IS, indoxyl glucuronide, and kynurenine, as well as phenolic uremic toxins like PS, disrupt the electron transport chain and associated dehydrogenases, affecting mitochondrial energy production (29). Furthermore, hippurate uremic toxins can upregulate dynamin-related protein 1 (Drp1), promoting mitochondrial fission (34). Exposure to TMAO reduces both LEAK (substrate-dependent) and OXPHOS (oxidative phosphorylation-dependent) mitochondrial respiration of pyruvate, affecting mitochondrial function (20). When mitochondrial structure and function are compromised, they become a primary source of ROS and RNS, thereby amplifying oxidative stress and activating innate immunity. This suggests that appropriate mitochondrial-targeted antioxidant strategies may reduce the risk of CKD by alleviating oxidative damage and modulating immune dysregulation (60). However, the intrinsic relationships among oxidative stress, mitochondrial dysfunction, uremic toxins, and immune disturbances have yet to be comprehensively delineated.

### 4.2 Endothelial dysfunction triggers an immune response to barrier injury

Endothelial dysfunction is common in moderate to advanced chronic kidney disease patients (61), with biomarkers including ICAM-1, VCAM-1, ADMA, ET-1, and IL-6 (62). Endothelial cell junctions include tight junctions (TJ), adherence junctions, and gap junctions, collectively maintain cellular homeostasis. In CKD, these cellular junctions may be impaired (63). Uremic toxins act through this mechanism, disrupting the intestinal cell barrier in the “kidney to gut” pathway and subsequently triggering an immune response to barrier disruption.

Uremia can affect tight junction protein ZO-1 and adherence junction protein VE-cadherin. Proteins like p120, β-catenin, and plakoglobin play important roles in regulating VE-cadherin (63). This pathological process is closely linked to immunological mechanisms. Indole uremic toxins, represented by IPA, mediate the pregnane X receptor (PXR) to reduce intestinal permeability, leading to endothelial dysfunction (39). Animal experiments have demonstrated that PXR-deficient mice exhibit “leaky” gut physiology (64). Indole and IAA can act on mucin genes, which play a role in maintaining epithelial function (57). Both IAA and IS can elevate tissue factor (TF) expression in human endothelium, increase TF’s procoagulant activity, and affect endothelial anticoagulant function. This process essentially initiates a pathological progression in which procoagulant activity becomes deeply intertwined with innate immune responses. Besides directly elevating TF, uremic toxins like kynurenine can increase TF via the AhR-TF/PAI-1 pathway. An imbalance in AhR signaling disrupts immune tolerance to commensal microbiota by skewing the Th17/Treg cell ratio in the intestinal lamina propria. IS can also inhibited the HIF/interleukin-10/vascular endothelial growth factor signaling pathway, then affected epithelial function (39). In this process, IL-10 and others involved are immune regulatory factors. Activation of the renin-angiotensin-aldosterone system (RAAS)/transforming growth factor-β (TGF-β) pathway can initiate its dysfunction (31, 42). Homocysteine leads to the degradation of intestinal epithelial TJs by inhibiting the expression of thrombomodulin and the binding of tissue plasminogen activator to endothelial cells, as well as stimulating the expression of oxidative and inflammatory factors, further contributing to endothelial dysfunction in CKD (31). In addition to affecting endothelial function, these mechanisms all reflect the interaction among immunity, inflammation and oxidative stress.

### 4.3 Full activation of the immune effector phase

**Figure 4:**
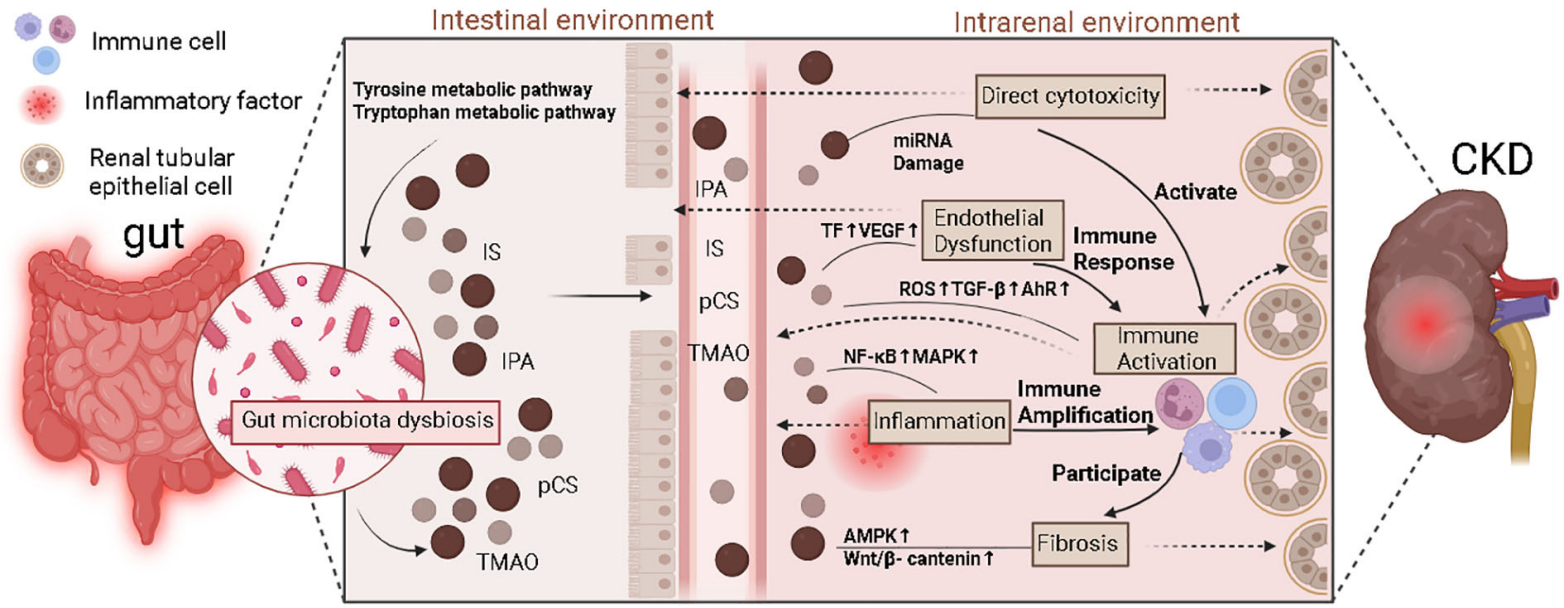
The mechanism of action of gut microbiota metabolites related to immunity in chronic kidney disease. In the intestinal environment, dysbiosis of the gut microbiota leads to an increase in the production of uremic toxins such as pCS, IPA, and TMAO. These metabolites, in conjunction with inflammatory factors released by immune cells, affect renal tubular epithelial cells through tyrosine and tryptophan metabolic pathways. In the intracellular environment, multiple signaling pathways (NF-κB, MAPK, AMPK, Wnt/β-catenin) are activated, accompanied by elevated ROS, TGF-β, AhR, and miRNA damage, thereby activating the immune response. In the renal environment, immune activation further participates in and amplifies the inflammatory response and fibrosis process, and simultaneously aggravates renal injury through mechanisms such as direct cytotoxicity, upregulation of transcription factors (TF) and VEGF, ultimately promoting the progression of CKD.

Immune dysfunction is highly implicated in the development of CKD and may be a key factor in this process (65). This process involves both innate and adaptive immune responses (**Figure 4**).

In the “kidney to gut” direction, existing research indicates that CKD can induce macrophages to activate immune responses. Uremic toxins like IS can cause renal tubular cells to release chemokines (*e.g*., ICAM-1, MCP-2, osteopontin, endothelin-1) and TGF-β1, promoting the proliferation of immune cells like macrophages. The pCS can also induce macrophage activation and inhibit the antibacterial immune response of LPS-induced macrophages (66). Besides macrophages, monocytes can also be affected by uremic toxins. The pCS inhibit the antibacterial immune response of LPS-induced monocytes (7). Similarly, TMAO can modulate VCAM-1 expression, promoting monocyte adhesion and exhibiting an immune response (57). Beyond the aforementioned immune cells, ILC2s originating from other organs such as the kidney migrate to the small intestine, where they adapt to the local microenvironment via the retinoic acid signaling pathway and acquire small intestine-specific phenotypes and functions, playing a critical role in combating helminth infections (*e.g*., Strongyloides ratti).Although RNA velocity analysis of kidney-derived ILC2s migrating into the small intestine suggests a progressive shift from a “kidney program” toward a “small intestine program” in effector ILC2s, definitive confirmation of the adaptive capacity of progeny ILC2s requires additional validation at the single-cell clonal resolution (67). Current research on adaptive immune responses in this field remains relatively limited, potentially indicating a knowledge gap.

In the “gut to kidney” direction, in terms of innate immune response, new research indicates that ILC3s, under the regulation of the CXCR6/CXCL16 axis, increase PD-1 expression and IL-17A production, and enhance the JAK2/STAT3/RORγt/IL-17A pathway by inhibiting IL-23R endocytosis (68). In terms of adaptive immune response, CKD can activate AhR in intestinal immune cells, thereby stimulating immunoregulatory T cells and activating immunity; I3A and ILA have this effect (39). Additionally, pCS can increase the level of undifferentiated CD8+ T cells, and H_2_S can inhibit the cytolytic activity of T lymphocytes (66). The pCS also have multiple immune effects, such as decreasing IFN-γ-producing Th1 cells, inhibiting the proliferation of CD43+ B cell progenitors *in vitro*, and directly or indirectly (by increasing respiratory burst and phagocytosis) reducing peripheral B cells. It is noteworthy that the documented influence of pCS on B cells is described through relatively indirect mechanisms. Given that the respiratory burst and phagocytic functions are primarily mediated by myeloid cells, a definitive causal relationship between these processes and B cell depletion has yet to be established. Furthermore, although evidence exists for the relationship between uremic toxins and the immune system, the specific molecular mechanisms by which PBUTs affect immune responses are not fully elucidated and require further research (69).

### 4.4 Oxidative stress and inflammation-driven immune amplification

Chronic inflammation is a key pathological feature of CKD, and oxidative stress is one factor leading to inflammation. The inflammatory response associated with the CKD—rather than the direct effects of uremic toxins on adaptive immunity—is the underlying cause of humoral immune dysfunction (70). The pathways involved in oxidative stress and pro-inflammation by uremic toxins play roles in both the “gut to kidney” and “kidney to gut” pathways.

Oxidative stress is caused by an imbalance between the production of reactive oxygen species (ROS) and the body’s antioxidant capacity (71). Common uremic toxins like IS and pCS can activate NADPH oxidase Nox4 to promote ROS generation (29). Simultaneously, these uremic toxins interfere with the body’s antioxidant capacity, downregulating glutathione peroxidase (GPx) activity and reducing the level of the antioxidant plasma glutathione. Furthermore, the significant correlation between IAA and the oxidative stress marker malondialdehyde further confirms the role of uremic toxins in oxidative stress (20).

NF-κB signaling is a key mediator of renal inflammation in CKD (72). NF-κB signaling can be directly activated by uremic toxins; phenolic uremic toxins can trigger NF-κB nuclear translocation to induce inflammation in CKD (29). Sulfate can also stimulate NF-κB mRNA expression (57). Uremic toxins can upregulate the NF-κB pathway by inducing ROS and activating the aryl hydrocarbon receptor (AhR). IAA can modulate the AhR/p38 MAPK/NF-κB pathway, increasing COX-2 expression (39). Similarly, IS can induce NF-κB activation via the ROS/NF-κB/p53 pathway. Sulfate can also enhance leukocyte activity *in vitro* via the ROS/JNK/NF-κB pathway to promote inflammation (57). Besides AhR and ROS, other pathways can mediate the NF-κB pathway, typically such as indole uremic toxins triggering JNK/MAPK/NF-κB signaling (31). The AhR can directly increase the expression of inflammatory markers like ICAM-1 and VCAM-1 (30), and can also elevate levels of CYP1A1, CYP1A2, and CYP1B1. When CYP1A1 metabolizes endogenous and exogenous substances, it can cause inflammatory responses (39). However, existing studies have demonstrated that activation of the AhR can protect intestinal barrier function by inducing IL-22 production, revealing the dual regulatory nature of AhR. This paradox suggests that inhibiting AhR to alleviate renal injury may inevitably compromise the protective immune barrier of the intestine, thereby undermining the clinical application of the gut-kidney axis theory (73). MAPK is also a common pathway activating oxidation and inducing inflammation. The MAPK pathways mediated by extracellular signal-regulated kinases (ERK1/2), p38 MAPK, and JNK represent non-canonical pathways activated by TGF-β1 and have pro-inflammatory effects (74). The uremic toxin IS can upregulate E-selectin via the p38 and p42/p44 MAPK pathways, mediating the recruitment of inflammatory leukocytes (29). It is worth noting that studies indicate IRAK4 can regulate MAPK (p38) signaling, promoting inflammatory responses (75). Similarly, ROS can also mediate the p38 MAPK pathway; IxS triggers inflammation via the ROS/p38 MAPK pathway (57). Additionally, pCS activate inflammation through another MAPK pathway, namely the intracellular pERK MAPK pathway (29). The JAK/STAT pathway is associated with inflammatory responses (76). Studies show that both I3A and sulfate can lead to STAT3 phosphorylation, inhibiting STAT3 expression (39). NLRP3 activation promotes the cleavage of pro-cytokines, triggering their release, and these mature cytokines are central to inflammation (77). The water-soluble small molecule uremic toxin TMAO can promote inflammatory responses through the activation of the ROS-TXNIP-NLRP3 inflammasome (20). There is a complex interaction between Klotho and the AKT/Nrf2 pathway (78). Common uremic toxins like sulfate can downregulate Nrf2 to induce inflammation (49). TLRs mediate inflammatory signaling transduction pathways by activating the regulation of pro-inflammatory cytokines (79), and TMAO and indoxyl sulfate upregulate TLR4 gene expression (80). AGEs can also induce inflammation through the classical ligand RAGE signaling (31). Beyond the pathways mentioned above, uremic toxins such as IS, pCS, TMAO, homocysteine, and H_2_S can also directly release inflammatory cytokines like TNF-α, IL-6, IL-1β, and IL-18, promoting inflammation.

Given the intricate synergistic and compensatory interactions among the aforementioned signaling pathways, intervention strategies targeting a single node are insufficient to fully reverse the chronic inflammatory microenvironment in the context of CKD.

### 4.5 Fibrotic remodeling in the immune microenvironment

Renal fibrosis is the core pathological process in the progression of CKD and constitutes a dysregulated tissue repair response with profound involvement of the immune system. It is characterized by excessive deposition of extracellular matrix (ECM), primarily mediated by myofibroblasts. These myofibroblasts originate from diverse sources, including fibroblasts, pericytes, epithelial cells, endothelial cells, and macrophages through respective transition processes. These transitions can be identified by the expression of alpha-smooth muscle actin (α-SMA) (81).

Multiple mechanisms collectively drive the development of renal fibrosis. EMT is highly prevalent in this process. Various inflammatory mediators and uremic toxins promote the EMT process and exacerbate renal fibrosis by activating the AhR signaling pathway. TGF-β signaling promotes the activation and proliferation of myofibroblasts and the production of ECM (82). Uremic toxins like IS and pCS induce EndMT primarily through TGF-β signaling and Smad-mediated canonical and non-canonical pathways (83). CKD patients, under the influence of the uremic toxin IS, exhibit upregulation of the TGF-β/Smad signaling, promoting the expression of α-SMA and ECM proteins, leading to renal fibrosis (29, 84). Similar to IS, pCS can also activate the TGF-β1/Smad pathway, reduce E-cadherin expression, and induce renal tubular EMT (29). RAAS is one mechanism of CKD progression. IS and pCS significantly activate the intrarenal RAAS by increasing the expression of renin, angiotensinogen, and AT1 receptors, and decreasing the expression of AT2 receptors *in vitro* and *in vivo*, inducing a phenotypic shift resembling EMT in renal tubules (85). The miR-29a-5p/GSAP/Notch1 pathway can also induce EndMT (86). The potential mechanisms of PMT remain unclear. Research by Xu et al. suggests that AMPK regulation of the PGC1α-CPT1A and HIF1α-HK2 pathways helps suppress PMT (87). Studies by Lin et al. show that fibroblast growth factor-23 (FGF-23) is positively correlated with IS serum levels and induces FMT transformation (88). Macrophages contribute to renal fibrosis through the tyrosine kinase Src, which activates both the TGF-β1/SMAD3 and JAK/STAT pathways to promote MMT (89). Additionally, Wnt/β-catenin is an important signaling pathway mediating renal fibrosis (90). Recent research has confirmed that the gut-derived toxin MMA can stimulate the Wnt/β-catenin pathway, causing renal tubules to exhibit significantly altered fibrosis (6). IAA also stimulates glomerulosclerosis and fibrosis by inducing COX-2 (7).

**Figure 5:**
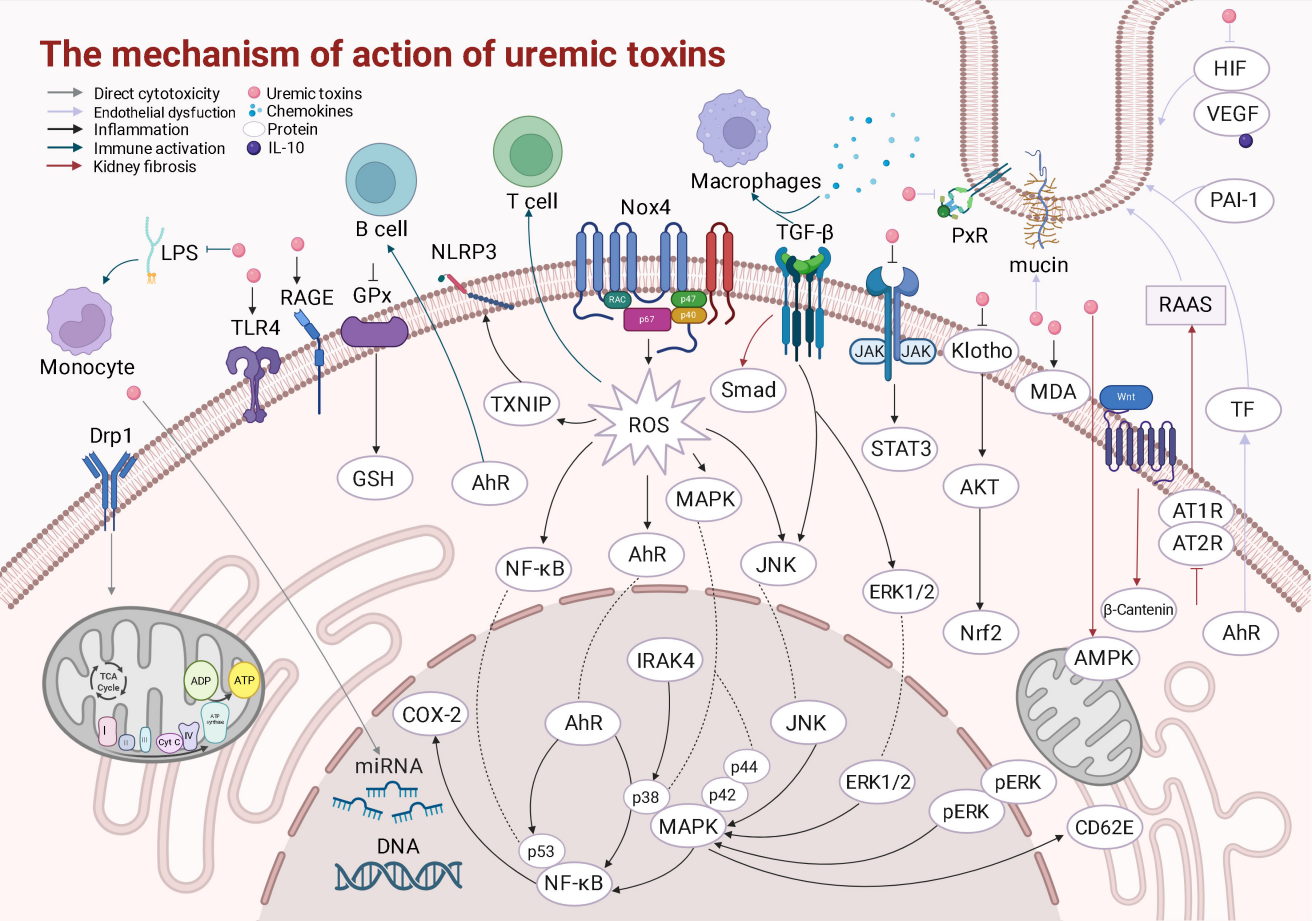
Uremic toxins accelerate CKD progression through different mechanisms. Disruption of the electron transport chain and associated dehydrogenases affecting mitochondrial energy, along with DNA methylation, exert direct cytotoxicity. PXR, mucin genes, TF, and the AhR-TF/PAI-1 and HIF/IL-10/VEGF pathways are associated with endothelial dysfunction. ROS, GPx, and malondialdehyde are related to oxidative stress. NLRP3, TLR4, NF-κB, MAPK, JAK/STAT, and RAGE promote inflammatory responses. Uremic toxins can influence macrophages, monocytes, T lymphocytes, and B lymphocytes through various pathways to activate immune responses. Renal fibrosis occurs via the TGF-β/Smad and Wnt/β-catenin pathways, leading to FMT, PMT, EMT, EndMT, and MMT.

It’s worth mentioning that many of the aforementioned pathways related to renal fibrosis have only been studied in animal models, and the association of some pathways with uremic toxins is not well explained. These fibrotic mechanisms are intertwined with the direct cytotoxicity, pro-inflammatory and oxidative stress effects, endothelial dysfunction, and immune-modulatory actions, collectively accelerating the progression of CKD (**Figure 5**).

## 5 Discussion and conclusion

CKD progression is closely linked to gut dysbiosis and uremic toxin accumulation. The present review systematically delineates the gut–kidney axis as a bidirectional, immunometabolic crosstalk network that profoundly influences the progression of chronic kidney disease (CKD). The accumulating evidence unequivocally establishes that gut microbiota dysbiosis—characterized by reduced α−diversity, depletion of butyrate−producing commensals (*e.g.*, *Faecalibacterium prausnitzii*, *Roseburia* spp., *Bifidobacterium*), and enrichment of pathogenic or conditionally pathogenic taxa harboring urease, tryptophanase, and tyrosine phenol−lyase—is a consistent hallmark of CKD across human cohorts and animal models. These compositional shifts translate into functional derangements: overproduction of protein−bound uremic toxins (indoxyl sulfate, p−cresyl sulfate, phenylacetylglutamine) and non−protein−bound toxins (trimethylamine N−oxide, asymmetric dimethylarginine) via the tryptophan, tyrosine, arginine, and advanced glycation end−product pathways. Dysbiosis (characterized by reduced microbial diversity and increased pathogen abundance) may facilitate toxin production, wherein these toxins—through induction of cytotoxicity, endothelial dysfunction, oxidative stress, inflammation, and fibrosis—drive an immune-centric vicious cycle, thereby exacerbating cardiovascular complications.

A central mechanistic insight emerging from this synthesis is that uremic toxins do not act through a single pathway but rather orchestrate a multilayered pathogenic network. They directly disrupt intestinal tight junctions (*e.g.*, ZO−1, occludin), increase gut permeability, and facilitate endotoxin translocation, thereby initiating systemic low−grade inflammation. At the renal level, these toxins induce direct cytotoxicity (mitochondrial dysfunction, DNA damage, epigenetic alterations), endothelial dysfunction (via VCAM−1, ICAM−1, tissue factor), and activation of both innate (macrophages, ILC3s) and adaptive (Th1/Th17/Treg dysregulation, B−cell modulation) immune responses. Critically, the NF−κB, MAPK, JAK/STAT, AhR, and NLRP3 inflammasome pathways serve as converging hubs that amplify oxidative stress and inflammation, ultimately driving epithelial–mesenchymal transition, myofibroblast activation, and irreversible renal fibrosis.

Despite the robustness of this conceptual framework, several critical gaps and caveats must be acknowledged. First, causality remains elusive. Most human studies are cross−sectional, unable to distinguish whether dysbiosis drives CKD progression or merely reflects the uremic milieu. Animal models, while informative, often employ acute or supra−physiological toxin loads (*e.g.*, adenine−induced nephropathy) that do not fully recapitulate the slow, multifactorial progression of human CKD. Germ−free or gnotobiotic transplantation studies directly proving that patient−derived microbiota accelerate renal fibrosis are still limited. Second, taxonomic heterogeneity and lack of reproducibility plague the field. Discrepancies—such as the opposing directionality of Prevotella at genus versus species level—highlight the inadequacy of 16S rRNA sequencing for functional inference. Strain−level resolution, metagenomics, and metabolomics are urgently needed to move beyond descriptive catalogues. Third, confounding variables (diet, medications including proton pump inhibitors and antibiotics, dialysis modality, residual renal function) are rarely rigorously controlled, potentially inflating spurious associations. Fourth, the classification of uremic toxins is conceptually muddled. Including host−derived cytokines (TNF−α, IL−6) as “middle molecule toxins” conflates microbial products with host immune effectors, obscuring therapeutic targeting. Moreover, some molecules (*e.g.*, spermidine, spermine) are paradoxically decreased in CKD, challenging their designation as true “toxins.” Fifth, the dual role of certain pathways—most notably the AhR—presents a therapeutic paradox: AhR activation contributes to renal injury and inflammation, yet AhR signaling in the gut is essential for maintaining barrier integrity via IL−22. Indiscriminate AhR inhibition might thus worsen intestinal permeability and endotoxemia. Sixth, the overwhelming majority of mechanistic data derive from animal models, and translation to humans remains unproven. Many signaling interactions (*e.g.*, IRAK4−MAPK, miR−29a−5p/GSAP/Notch1) have not been validated in CKD patients.

From a clinical perspective, the gut–kidney axis offers actionable opportunities but also significant challenges. Current interventions—dietary protein restriction, prebiotics/probiotics, oral adsorbents (*e.g*., AST−120), and emerging microbial enzyme inhibitors—have shown only modest efficacy in clinical trials. This may reflect the redundancy and compensatory plasticity of the gut microbial ecosystem: targeting a single bacterial enzyme or a few taxa is unlikely to durably alter toxin production. Moreover, the substantial inter−individual variability in baseline microbiota composition argues for personalized rather than one−size−fits−all strategies.

In conclusion, the gut–kidney axis represents a pivotal bidirectional mechanism in CKD progression, wherein gut dysbiosis and uremic toxin overproduction drive a self−perpetuating cycle of inflammation, oxidative stress, immune dysregulation, and renal fibrosis. To translate this concept into clinical benefit, the field must move beyond descriptive taxonomy toward functional, causal investigations—prioritizing longitudinal multi−omics cohorts, mechanistic validation using defined microbial consortia or enzyme inhibitors, and personalized interventions such as next−generation probiotics, postbiotics, or dietary modulation. Resolving pathway paradoxes (*e.g*., dual roles of AhR) and rigorously controlling confounders will be essential. Ultimately, harnessing the gut–kidney axis for therapy requires integrated immunometabolic strategies, offering hope for slowing CKD progression in millions of patients.
